# Effects of recumbency and tube current on image quality of canine elbow computed tomography

**DOI:** 10.3389/fvets.2026.1914425

**Published:** 2026-09-04

**Authors:** S. R. Bellekom, M. Dekkers, M. Beukers

**Affiliations:** IVC Evidensia Referral Hospital Hart van Brabant, Waalwijk, Netherlands

**Keywords:** ALARA, canine elbow dysplasia, computed tomography, image quality, medial coronoid process, patient positioning, SNR (signal noise ratio), tube current

## Abstract

**Background:**

Elbow dysplasia is a leading cause of forelimb lameness in medium- to large-breed dogs, and computed tomography (CT) is the imaging modality of choice for assessment of the medial coronoid process. Image quality depends on patient positioning and tube current, but their effects have not been systematically compared.

**Aims:**

To compare the image quality of two canine elbow CT positioning protocols (dorsal recumbency: head in neutral position, both elbows scanned together vs. lateral recumbency: head/neck flexed caudally, elbows scanned separately), and to evaluate the influence of tube current and its interaction with these protocols.

**Methods:**

Prospective experimental cadaver study on twenty canine cadavers (40 elbows). All cadavers were scanned in both positions at 150 mA; a subgroup of eight was additionally scanned at 300 mA. Quantitative outcomes were signal- and contrast-to-noise ratios in bone- and soft-tissue-kernels. Qualitative outcomes, assessed by three observers, were paired-preference reads of four criteria: bone conspicuity, soft-tissue conspicuity, and artifacts in bone- and soft-tissue window. Inter- and intra-observer agreement were quantified for the bone conspicuity criterion.

**Results:**

Lateral recumbency produced significantly higher signal- and contrast-to-noise ratios than dorsal recumbency for all four outcomes (all *p* < 0.001 at 150 mA). Doubling the tube current from 150 to 300 mA did not produce a statistically significant within-recumbency improvement (all *p* ≥ 0.058). Lateral recumbency at 150 mA remained superior to dorsal at 300 mA on all four outcomes (*p* = 0.002 to 0.013). Qualitatively, observers almost unanimously preferred lateral recumbency for the artifact criteria and for soft-tissue conspicuity (99–100% lateral). For bone conspicuity, 31% of paired comparisons were ties; lateral was preferred in 60.6% of non-tie ratings (*p* = 0.008).

**Conclusion:**

Lateral recumbency, scanning the elbows separately with head and neck flexed caudally, provides superior objective image quality, and increased tube current does not compensate for suboptimal positioning. The observer-perceived advantage for bone conspicuity was inconsistent. For routine orthopedic CT focused on the medial coronoid process, dorsal recumbency remains a reasonable choice; lateral recumbency should be preferred when artifact reduction or soft-tissue evaluation is clinically important.

## 1 Introduction

Elbow dysplasia (ED) is a leading cause of forelimb lameness in young, medium- to large breed dogs (1). Reported ED prevalence varies widely by population: O’neill et al. (1) reported elbow joint disease in below 1% of UK primary-care dogs, of which 30.84% were attributable to ED; prevalence reaches 17–70% in predisposed breeds and screening populations (2), with bilateral involvement in approximately 88% (3).

The term “elbow dysplasia” encompasses a group of developmental abnormalities of the elbow joint, namely joint incongruency, osteochondrosis (OC) and/or osteochondritis dissecans (OCD), medial coronoid process disease with or without fragmentation (MCD), and ununited anconeal process (UAP), which may occur alone or in combination (2, 3).

Computed tomography (CT) is a non-invasive modality widely used to evaluate canine ED, and is considered the imaging modality of choice for assessing the medial coronoid process (4, 5). In a recent screening-population study of 424 elbow joints that used CT as the reference standard, radiography yielded a sensitivity of 65%, specificity of 93%, positive predictive value of 37%, and negative predictive value of 98% for the detection of medial coronoid disease, supporting CT as the preferred modality when available (6). Compared with conventional radiography, CT offers detailed cross-sectional anatomy without superimposition of surrounding structures, adjustable grayscale window settings, superior contrast resolution, and the ability to generate multiplanar reconstructions (4, 5). Its diagnostic role continues to expand, and recent work has shown that contrast-enhanced protocols can provide additional information on periarticular soft-tissue changes in dogs with elbow disorders (7).

Accurate assessment of the elbow joint depends on adequate image quality, which in CT is determined primarily by spatial resolution, contrast resolution and image noise (8). Spatial resolution is influenced by the focal spot size of the X-ray tube, detector element size, helical pitch, and reconstruction kernel. Contrast resolution is influenced by the tube current-time product (mAs), tube voltage (kVp), slice thickness, and reconstruction kernel (9).

An optimal elbow CT protocol should minimize both image noise and artifacts. Artifacts are image features that do not correspond to real anatomy and may obscure or mimic pathology (10). In canine elbow CT, the most clinically relevant artifacts are beam-hardening and photon-starvation artifacts (10, 11). Beam-hardening artifacts appear as streaks and dark bands and arise from the preferential attenuation of low-energy photons as the X-ray beam passes through dense or thick structures such as skull, cervical spine and contralateral limb. Photon-starvation artifacts arise when too few photons reach the detector after traversing highly attenuating regions, and they manifest as streaks aligned with the direction of greatest attenuation (10, 11). The severity of both artifacts depends on patient positioning, which determines the structures lying in the beam path at the level of the elbow joints, and on tube current, as a higher mA provides more photons to traverse attenuating tissue (10, 11).

Several positioning protocols are used for canine elbow CT. In a commonly used approach, the patient is positioned in dorsal recumbency with both thoracic limbs extending cranially and the head in a neutral position on the CT table, resulting in the head and neck being included in the scan field. Alternatively, both elbows can be scanned together in lateral recumbency while still including the head and neck in the scan field (12). The same acquisition can then be reconstructed to include the shoulders and cervical spine, which can be clinically valuable because forelimb lameness is often difficult to localize to the elbow alone (5, 12). Other protocols displace the head and neck out of the scan plane (2, 5–7). These can be performed in sternal or lateral recumbency, with either both thoracic limbs or a single thoracic limb extended cranially. Excluding the head and neck reduces the effective tissue thickness traversed by the X-ray beam at the level of the elbow, thereby reducing beam-hardening and photon-starvation artifacts (10, 11).

The primary aim of this study was to compare the image quality of elbow CT scans in dogs acquired in dorsal recumbency, with both thoracic limbs extended cranially and the head in a neutral position, versus lateral recumbency, with a single thoracic limb extended cranially and the head and neck flexed caudally. The secondary aim was to evaluate the influence of tube current (mA) on image quality in each position. We hypothesised that, at comparable mA settings, lateral recumbency would produce fewer beam-hardening and photon-starvation artifacts than dorsal recumbency, and that the magnitude of this positioning effect would diminish as mA increased.

## 2 Materials and methods

### 2.1 Study design

This was a prospective, experimental cadaver study on twenty canine cadavers; both elbow joints of each cadaver were scanned, yielding forty elbow joints for analysis. All twenty cadavers were scanned in both dorsal and lateral recumbency at 150 mA to address the primary aim (effect of recumbency on image quality). Eight of the twenty cadavers were additionally scanned in both recumbencies at 300 mA to address the secondary aim (effect of tube current on image quality and its interaction with recumbency).

### 2.2 Study population

Fresh canine cadavers were obtained from one veterinary hospital (*Evidensia Referral Hospital Hart van Brabant, Waalwijk, The Netherlands*) immediately after euthanasia, performed for reasons unrelated to this study. All cadavers were scanned within 1 h of euthanasia; no intervening refrigeration or freezing was required.

Inclusion criteria were skeletal maturity (≥1 year of age) and a body weight of 23–50 kg. All included cadavers met the body-weight inclusion criterion. Because this study was designed to evaluate image quality rather than to detect or characterize elbow pathology, no exclusion criteria relating to the presence of elbow disease were applied. The only exclusion criterion applied was previous orthopedic surgery of the thoracic limb, because metallic implants produce beam-hardening and streak artifacts that are distinct from the positioning-related artifacts under investigation.

Cadaver inclusion was verified by a European College of Veterinary Diagnostic Imaging (ECVDI) third-year resident (S. B.). Breed, age, sex and body weight were recorded for each cadaver.

Written informed consent was obtained from all owners at the time of admission for the use of their animal’s cadaver in research.

### 2.3 Computed tomography examination

**Figure 1:**
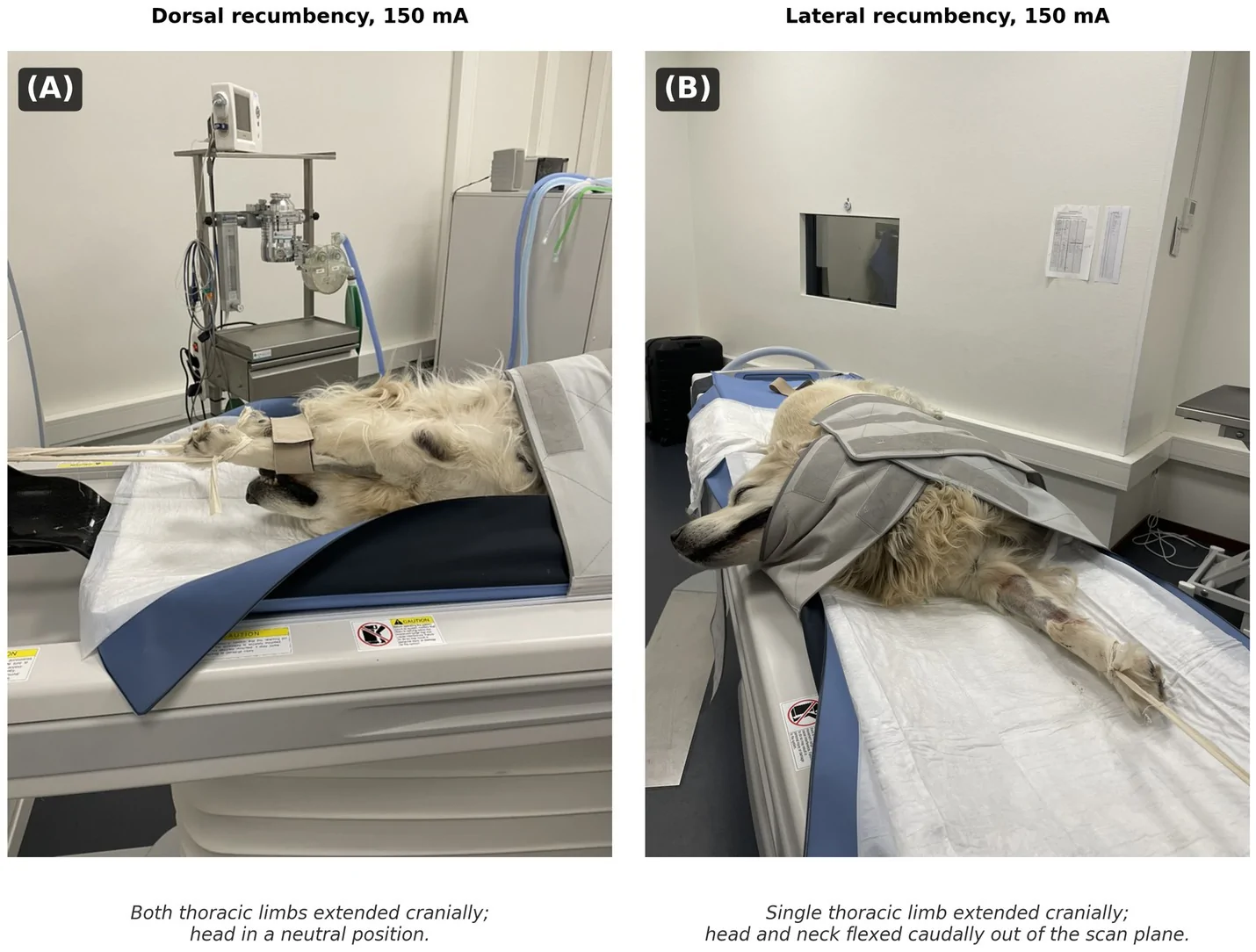
Patient positioning of the canine patient for elbow computed tomography, illustrating the two protocols evaluated. **(A)** Dorsal recumbency: Both thoracic limbs extended cranially and the head in a neutral position on the CT table. **(B)** Lateral recumbency: a single thoracic limb extended cranially and the head and neck flexed caudally, out of the scan plane at the level of the elbow. The positioning aid visible over the head and neck in **(B)** was not within the scan field during CT acquisition. In lateral recumbency, each elbow was scanned in a separate acquisition with the corresponding limb extended.

All examinations were performed using an 80-slice helical CT scanner (*Aquilion Lightning, Canon Medical Systems, Europa BV, Amstelveen, the Netherlands*). Each cadaver was scanned in two recumbencies. In dorsal recumbency, the cadaver was placed on its back with both thoracic limbs extended, separating the elbow joints and humeral diaphysis from the shoulders in the *z*-axis of the acquisition. In lateral recumbency, the two elbows were scanned in separate acquisitions, each with a single thoracic limb extended cranially and the head and neck flexed caudally, reducing the volume of tissue traversed by the X-ray beam at the level of the elbow joint (Figure 1).

The scan range extended from 1 cm proximal to the olecranon to approximately 3 cm distal to the radial head. A tube current of 150 mA was used in all twenty cadavers; the eight-cadaver subgroup additionally received acquisitions at 300 mA. These settings were based on tube currents reported for canine elbow CT in the peer-reviewed literature, which range from approximately 100 mA to 350 mA depending on scanner and kilovoltage (2, 6, 7). 150 mA at 100 kVp corresponds to the standard clinical protocol used at our institution for canine elbow CT on this scanner, established in consultation with the manufacturer’s clinical application specialist. During protocol development an intermediate tube current of 225 mA was also acquired but was not included in the formal analysis, as no visible difference from 300 mA was appreciated on preliminary review; 300 mA was retained for the higher-dose condition as a clean two-fold factor for statistical comparison. All other acquisition parameters remained constant: tube voltage 100 kVp, 0.638 pitch, slice thickness 0.5 mm, matrix 512 × 512, field of view 199.2 mm and focal spot of 0.9 × 0.7 mm. In dorsal recumbency, the acquisition covered both elbow joints; each elbow was then reconstructed as a separate volume using an individual, elbow-centred display field of view of 199.2 mm, matched to the display field of view used for the single-elbow acquisitions in lateral recumbency, so that pixel size and image-analysis conditions were equivalent between recumbencies. In lateral recumbency, each elbow was acquired and reconstructed with the same display field of view. Pixel size was therefore consistent (0.39 × 0.39 mm) across all acquisitions of a given elbow, so paired region-of-interest measurements were based on identically sampled voxels. Images were reconstructed at the institutional default settings, using bone (FC30) and soft-tissue (FC08) reconstruction kernels.

DICOM images were reviewed in transverse reconstruction using MedDream (*Softneta UAB, Kaunas, Lithuania; version 8.7.0*) and Horos (version 4.0.1; Horos Project), with bone (window width 2000 HU, window level 400 HU) and soft-tissue (window width 400 HU, window level 40 HU) window setting.

### 2.4 Assessment of image quality

#### 2.4.1 Quantitative analysis

Quantitative image analysis was performed by the third-year ECVDI resident (S. B.). A single region of interest (ROI) was placed for each anatomical structure at a predefined axial level, using anatomical landmarks—specified further in detail below—to ensure that matched levels were used across positions and cadavers. The target ROI area was 50 mm^2^, with a circular shape where anatomy permitted. Where anatomy did not permit a circular ROI of this size, specifically the humeral cortex and the subcutaneous fat adjacent to the triceps, a smaller ROI of 20 mm^2^ was used. ROI shape was elliptical in these constrained regions, with the long axis oriented parallel to the cortical surface or along the fat plane. The same ROI size and shape were used for a given anatomical location across all acquisitions of a given elbow, so that paired comparisons were based on identically placed measurements. On bone-kernel images, ROIs were placed in ambient air outside the cadaver, in the humeral cortex, and in the triceps muscle. On soft-tissue-kernel images, ROIs were placed in ambient air, in the triceps muscle, and in the subcutaneous fat adjacent to the triceps. The mean attenuation (in Hounsfield units, HU) and standard deviation (SD) within each ROI were recorded. Image noise was defined as the SD of attenuation within the air ROI. Signal-to-Noise ratio (SNR) and contrast-to-noise ratio (CNR) were calculated using the following equations: (9, 10).


SNRbone=HUHC/SDAIR

SNRsoft tissue=HUTM/SDAIR

CNRbone=(HUHC–HUTM)/SDAIR

CNRsoft tissue=(HUTM–HUFAT)/SDAIR

Where HC denotes the humeral cortex, AIR the ambient air, TM the triceps muscle, and FAT the subcutaneous fat adjacent to the triceps.

#### 2.4.2 Qualitative analysis

Qualitative assessment was performed by three observers (M. B. and M. D., ECVDI-certified veterinary radiologists; S. B., third-year ECVDI resident). Each elbow was evaluated on four criteria: conspicuity of trabecular bone and the medial coronoid process in bone window; presence and severity of beam-hardening and photon-starvation artifacts in both bone and soft-tissue window; and conspicuity of muscles in soft-tissue window. Studies were anonymised, presented in randomised order, and intended to be viewed blinded to cadaver identity, left or right elbow, recumbency and (for the subgroup) tube current; condition labels in Horos were hidden. Complete blinding to recumbency was, however, not achievable, as partial visibility of the CT table within the imaged field allowed recumbency to be inferred from the image itself. Blinding to cadaver identity, elbow side and tube current was not affected. In the full group (*n* = 20) each elbow contributed one paired comparison of dorsal versus lateral recumbency at 150 mA. In the subgroup (*n* = 8), the four conditions per elbow (low-dose lateral recumbency (L150), high-dose lateral recumbency (L300), low-dose dorsal recumbency (D150) and high-dose dorsal recumbency (D300)) additionally generated four paired comparisons per window for the secondary analyses: dose effect within each recumbency (L150 vs. L300; D150 vs. D300), and recumbency effect at high dose (L300 vs. D300). Furthermore, a cross-comparison L150 vs. D300 was performed to test whether higher tube current could compensate for suboptimal positioning.

For each comparison, observers chose the image series with superior image quality or scored a tie if no difference was observed. Both groups were scored on two occasions 3 weeks apart, in re-randomised order. Based on preliminary review of the first-session preferences, the second session was restricted to bone-window conspicuity, the only criterion that demonstrated substantial inter-observer variability, allowing assessment of intra-observer agreement for this criterion.

#### 2.4.3 Statistical analysis

Statistical analyses were performed using JASP (*version 0.95.4 (Apple Silicon); JASP Team, University of Amsterdam, The Netherlands*).

For the recumbency analysis (*n* = 20), SNR and CNR were analysed using a linear mixed-effects model on log-transformed values, with recumbency as a fixed effect and cadaver identity as a random effect; estimates and 95% confidence intervals were back-transformed by exponentiation for presentation. For soft-tissue outcomes, the linear mixed-effects model produced singular-fit warnings; Wilcoxon signed-rank tests were therefore used as the primary inference for these outcomes. For the subgroup analyses (*n* = 8) of the tube-current effect within each recumbency, the recumbency effect at high dose, and the cross-comparison of lateral recumbency at 150 mA versus dorsal recumbency at 300 mA, Wilcoxon signed-rank tests were used.

Qualitative scores were summarised descriptively, and differences between conditions were tested using exact binomial sign tests on the non-tie scores. Inter- and intra-observer agreement on the bone-window conspicuity criterion were quantified using linearly weighted Cohen’s kappa, interpreted according to Landis and Koch (≤0.00 poor; 0.01–0.20 slight; 0.21–0.40 fair; 0.41–0.60 moderate; 0.61–0.80 substantial; 0.81–1.00 almost perfect) (13). Linear weighting was used because a three-category outcome (lateral/tie/dorsal) is ordinal with respect to the preference direction, and a lateral-vs-dorsal disagreement is more severe than an adjacent disagreement (lateral-vs-tie or tie-vs-dorsal).

## 3 Results

### 3.1 Study population

Twenty canine cadavers were included in this study, yielding forty elbow joints for analysis. Breeds represented were Labrador Retriever (*n* = 4), Boxer (*n* = 2), Malinois (*n* = 2), and one each of Bull Terrier, Golden Retriever, American Bulldog, German Shepherd Dog, American Bully Standard, Bouvier des Flandres, Border Collie, Greek crossbreed, Husky, American Staffordshire Terrier, mixed breed and Rhodesian Ridgeback. Sex distribution was 7 male intact, 4 male neutered, 5 female intact and 4 female neutered. Median age was 8.9 years (range 1–13.3) and median body weight was 34.9 kg (range 27–50). Since the cadavers were predominantly those of older, large-breed dogs, pre-existing medial coronoid disease and degenerative changes were subjectively observed on CT review in a substantial proportion of the elbows. Formal per-elbow pathology assessment was not undertaken, as characterisation of elbow pathology was not an aim of this study.

### 3.2 Quantitative image quality

#### 3.2.1 Effect of recumbency (full group, *n* = 20 cadavers, 40 elbows at 150 mA)

Lateral recumbency produced substantially higher SNR and CNR than dorsal recumbency in both bone- and soft-tissue-kernels. For bone-kernel outcomes, the linear mixed-effects model showed a highly significant effect of recumbency for both SNR [*F*(1,19) = 78.34, *p* < 0.001] and bone CNR [*F*(1,19) = 74.76, *p* < 0.001]. Back-transformed marginal means were 60.8 (95% CI 52.5–70.5) for lateral and 27.7 (95% CI 22.6–34.0) for dorsal recumbency for bone SNR – a 2.2-fold improvement—and 58.4 (95% CI 50.2–68.0) versus 26.5 (95% CI 21.4–32.7) for bone CNR, also a 2.2-fold improvement. For soft-tissue outcomes, Wilcoxon signed-rank tests were used as the primary inference. Lateral recumbency resulted in a significantly higher soft-tissue SNR (mean ± SD: 14.7 ± 5.5 lateral, 7.1 ± 2.6 dorsal; *W* = 53, *p* < 0.001) and CNR (43.3 ± 15.9 lateral, 21.2 ± 8.2 dorsal; *W* = 62, *p* < 0.001) than dorsal recumbency, with lateral preferred in 35 of 40 paired elbows for both outcomes.

#### 3.2.2 Effect of tube current and its interaction with recumbency (subgroup, *n* = 8 cadavers, 16 elbows)

The recumbency effect was preserved at the higher tube current. At 300 mA, lateral recumbency produced significantly higher values than dorsal recumbency for all four outcomes: bone SNR (mean 70.6 vs. 37.7; *W* = 5, *p* < 0.001), bone CNR (67.8 vs. 36.3; *W* = 5, *p* < 0.001), soft-tissue SNR (13.9 vs. 6.6; *W* = 11, *p* = 0.002), and soft-tissue CNR (41.6 vs. 19.9; *W* = 10, *p* = 0.001).

Doubling the tube current from 150 mA to 300 mA did not produce a statistically significant change in any outcome within either recumbency. Within lateral recumbency, none of the within-recumbency dose comparisons reached statistical significance (bone SNR and bone CNR *W* = 31, both *p* = 0.058; soft-tissue SNR and CNR *W* = 54, *p* = 0.495). The same pattern was observed within dorsal recumbency (bone SNR and bone CNR *W* = 31, *p* = 0.058; soft-tissue SNR *W* = 98, *p* = 0.130; soft-tissue CNR *W* = 95, *p* = 0.175).

In the pre-specified cross-comparison, lateral recumbency at 150 mA produced significantly higher values than dorsal recumbency at 300 mA for all four outcomes: bone SNR (*W* = 11, *p* = 0.002; mean 63.6 vs. 37.7), bone CNR (*W* = 11, *p* = 0.002; 61.1 vs. 36.3), soft-tissue SNR (*W* = 18, *p* = 0.008; 13.1 vs. 6.6), and soft-tissue CNR (*W* = 21, *p* = 0.013; 38.9 vs. 19.9). Doubling the tube current in dorsal recumbency therefore did not recover the image quality available from optimal positioning at half the dose.

### 3.3 Qualitative image quality

#### 3.3.1 Effect of recumbency (full group, *n* = 20 cadavers, 40 elbows at 150 mA)

**Figure 2:**
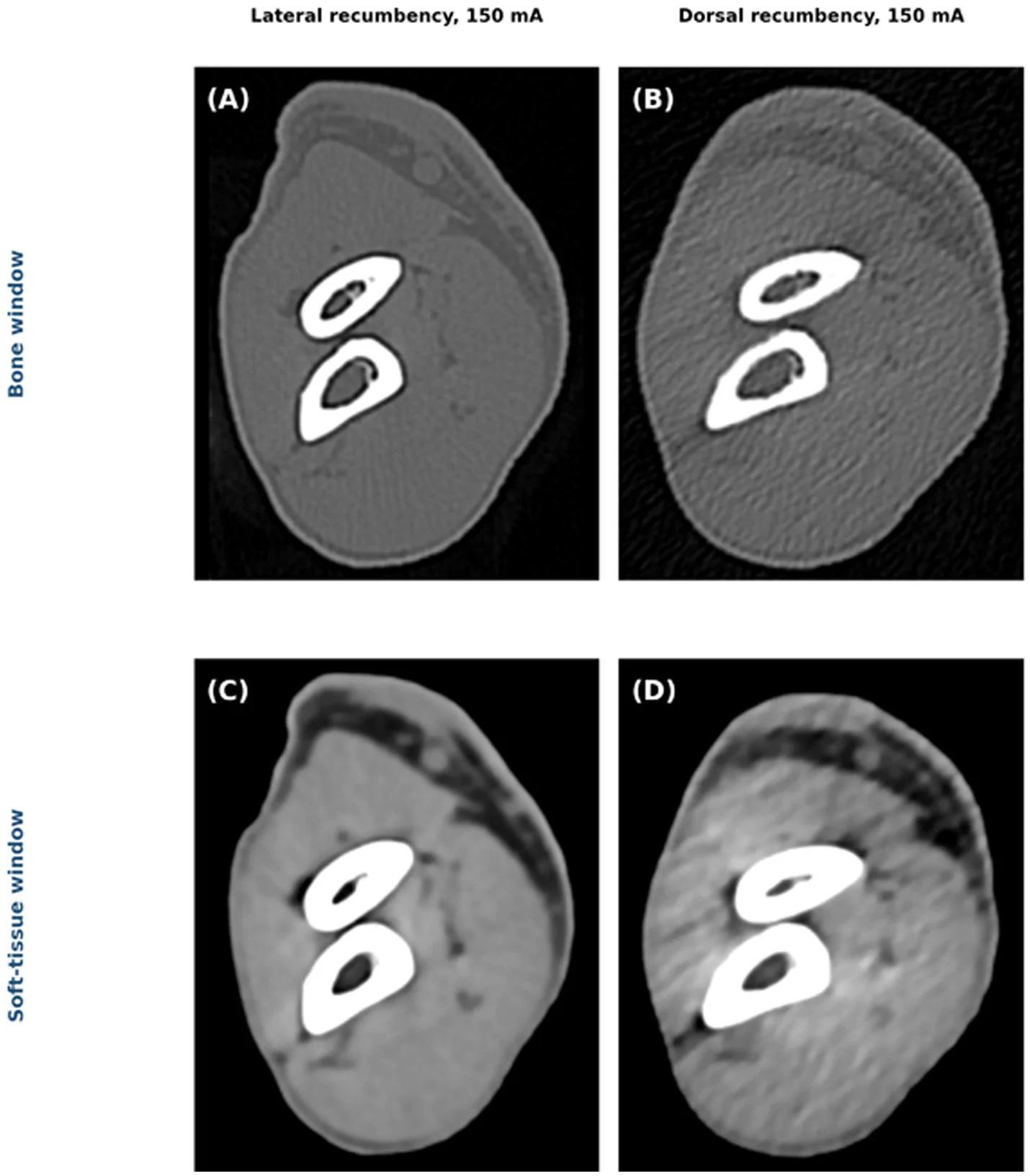
Transverse computed tomography images of one canine elbow (case 2 left elbow) at the level of the medial coronoid process, acquired at 150 mA in lateral recumbency **(A,C)** and dorsal recumbency **(B,D)**. **(A,B)** Bone window (W 2000/L 400). **(C,D)** Soft-tissue window (W 400/L 40). Beam-hardening and photon-starvation streak artifacts in this case are evidently more visible in the dorsal recumbency images **(B,D)**.

Three of the four criteria—bone-window artifacts, soft-tissue conspicuity, and soft-tissue artifacts—produced near-unanimous observer preferences in favour of lateral recumbency, with negligible tie rates (Figure 2). Across 120 observer-comparisons per criterion (single scoring session), lateral recumbency was rated superior in 119 (99.2%), 119 (99.2%), and 120 (100%) comparisons respectively, with 1 tie (0.8%) for bone artifacts and no ties for either soft-tissue criterion. Among non-tie ratings, lateral recumbency was preferred in essentially all comparisons (all *p* < 0.001). This near-unanimity across the three criteria at the first session was the basis for restricting the second scoring session to bone-window conspicuity. Inter- and intra-observer agreement was quantified only for the bone-window conspicuity criterion, because the other three qualitative criteria produced near-unanimous preferences with insufficient variance for meaningful kappa estimation; absolute agreement between observer pairs for these criteria was 95–100%.

**Figure 3:**
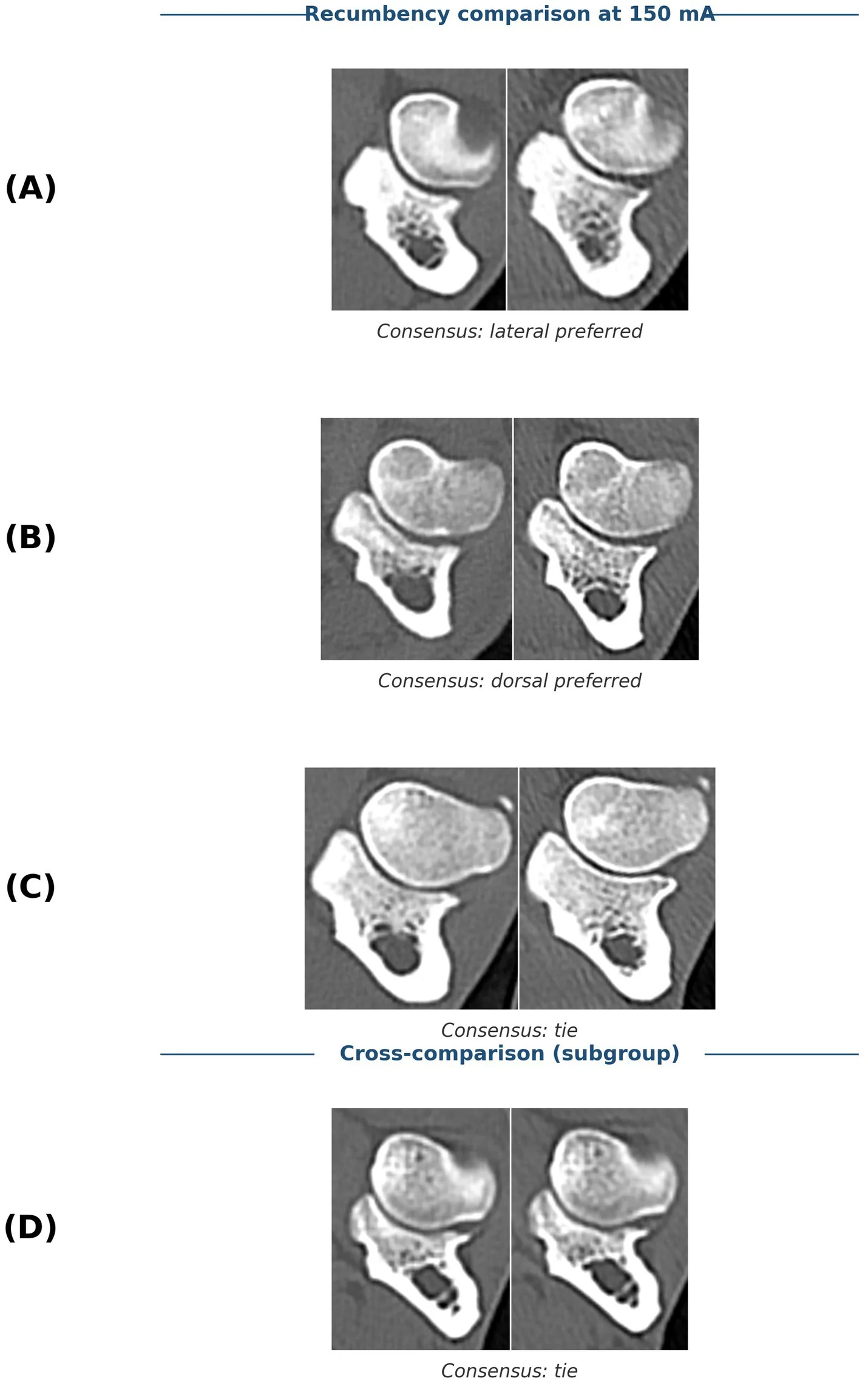
Bone-window transverse CT images (W 2000/ L 400) at the level of the medial coronoid process for four paired comparisons illustrating the diversity of subjective bone-conspicuity outcomes. In each panel, lateral recumbency is shown on the left and dorsal recumbency on the right. **(A)** Lateral preferred (case 6 right elbow). **(B)** Dorsal preferred (case 3 right elbow). **(C)** Rating was tie (case 8 right elbow). **(D)** Cross-comparison from the subgroup (case 1A right elbow): Lateral recumbency at 150 mA (L150) versus dorsal recumbency at 300 mA (D300)—observer rating was tie. The diversity of subjective outcomes for the same bone-window comparisons, set against consistent ~2-fold objective SNR/CNR differences in the same elbows, illustrates the partial dissociation between objective and subjective image quality discussed in the text.

For bone-window conspicuity, pooled across both scoring sessions (240 observer-comparisons), observer preferences were notably more variable. Lateral recumbency was rated superior in 100 comparisons (41.7%), dorsal recumbency in 65 (27.1%), and 75 (31.2%) were rated as ties (Figure 3). The high proportion of ties—nearly one in three paired comparisons—indicates that a substantial fraction of reads produced no detectable subjective difference between recumbencies, despite the quantitative SNR and CNR differences in the same elbows. Among non-tie ratings, lateral recumbency was preferred over dorsal more often than expected by chance (sign test: 100 lateral vs. 65 dorsal of 165 non-tie ratings, *p* = 0.008), but the modest non-tie margin (60.6% favouring lateral) and high tie rate together indicate a limited and inconsistent perceptual benefit of lateral recumbency for bone-window conspicuity. Inter-observer agreement for the bone-window conspicuity criterion was slight to moderate (weighted *κ* = 0.12–0.45 at session 1; 0.26–0.50 at session 2). Intra-observer agreement between sessions for the same criterion was slight to fair (*κ* = 0.11–0.35; absolute agreement 42.5–57.5%); by observer: S. B. *κ* = 0.19 (42.5% agreement), M. B. *κ* = 0.11 (42.5%), M. D. *κ* = 0.35 (57.5%).

#### 3.3.2 Effect of tube current and its interaction with recumbency (subgroup, *n* = 8 cadavers, 16 elbows)

For the artifact criterion in both windows, observer preferences were near-unanimous with low tie rates. Lateral recumbency at 300 mA was rated superior over dorsal at 300 mA in 100% of comparisons in both windows (no ties; *p* < 0.001 among non-tie ratings). The higher tube current was preferred within each recumbency: within lateral, L300 was preferred in 83.3% (bone) and 85.4% (soft-tissue) of comparisons with 16.7 and 14.6% ties, respectively, and no L150 preferences; within dorsal, D300 was preferred in 97.9% (bone) and 100% (soft-tissue) with 2.1 and 0% ties; all *p* < 0.001 among non-tie ratings. In the cross-comparison, lateral recumbency at 150 mA was strongly preferred over dorsal recumbency at 300 mA for artifact burden (93.8% L150 with 4.2% ties for bone, 2.1% for D300; 100% L150 with no ties for soft-tissue; both *p* < 0.001 among non-tie ratings).

For soft-tissue conspicuity, lateral recumbency was strongly preferred over dorsal at 300 mA (95.8% L300, no ties; *p* < 0.001 among non-tie ratings) and in the cross-comparison (95.8% L150, 2.1% tie, 2.1% D300; *p* < 0.001 among non-tie ratings). The higher tube current was strongly preferred within dorsal recumbency (93.8% D300, 6.2% tie; *p* < 0.001 among non-tie ratings). Within lateral recumbency, however, two-thirds of comparisons were rated as ties (33.3% L300, 66.7% tie, no L150 preferences). Among the 16 non-tie ratings, L300 was preferred unanimously (*p* < 0.001), but the high tie rate indicates that for most paired comparisons observers detected no subjective difference between doses once lateral positioning had been used.

Bone-window conspicuity again showed the greatest observer variability, with the highest tie rates of any criterion. Within lateral recumbency, the dose comparison was rated as a tie in 62.5% of comparisons (3.1% L150, 62.5% tie, 34.4% L300); within dorsal recumbency, the pattern was similar (1.0% D150, 63.5% tie, 35.4% D300). Among non-tie ratings, L300 and D300 were preferred in both cases (both *p* < 0.001), but the high tie rates indicate that for most paired comparisons observers detected no subjective difference between doses. For the recumbency comparison at high dose, preferences were distributed across all three categories (34.4% L300, 36.5% tie, 29.2% D300), with no significant preference detected among non-tie ratings (sign test, *p* = 0.61). Similarly, the cross-comparison showed a near-uniform distribution (35.4% L150, 31.2% tie, 33.3% D300; sign test among non-tie ratings, *p* = 0.90), indicating that observers could not distinguish bone conspicuity between lateral recumbency at 150 mA and dorsal recumbency at 300 mA. Inter-observer agreement for this criterion ranged from poor to moderate (weighted *κ* = −0.03 to 0.50 across sessions and observer pairs). Intra-observer agreement between sessions was variable (*κ* = −0.31–1.00; median 0.22); by observer: S. B. *κ* = −0.12 to 0.63 (median 0.20), M. B. *κ* = 0.29–1.00 (median 0.60), M. D. *κ* = −0.31 to 0.22 (median 0.04).

## 4 Discussion

In this prospective experimental cadaver study, lateral recumbency produced significantly higher signal- and contrast-to-noise ratios for both bone- and soft-tissue-kernels CT acquisitions of the canine elbow than dorsal recumbency. The benefit of lateral recumbency was approximately two-fold for all four quantitative outcomes, was preserved at both tube currents, and could not be compensated by doubling the tube current in dorsal recumbency. Subjective image quality assessments showed the same pattern for the artifact criterion in both windows and for soft-tissue conspicuity, where observers near-unanimously preferred lateral recumbency. For bone-window conspicuity, however, observers frequently perceived no difference between the recumbencies: nearly a third of paired comparisons were rated as ties and observer preferences for either recumbency were inconsistent, indicating that the large objective difference between recumbencies did not translate into a consistent perceptual advantage.

In dorsal recumbency, the X-ray beam at elbow level must traverse the contralateral thoracic limb and the adjacent head-and-neck tissues, both of which lie within the scan plane at that anatomical level. The dense, thick tissue in the beam path attenuates low-energy photons more strongly than high-energy photons, producing beam-hardening artifacts, and reduces the total photon flux reaching the detector, producing photon-starvation artifacts (10, 11). In lateral recumbency as used in this study, both the contralateral thoracic limb and the head and neck were positioned away from the imaged joint; the contralateral limb extended caudally along the body and the head and neck flexed caudally out of the scan plane. On general CT-physics grounds, this reduction in the volume of dense soft tissue and bone traversed by the X-ray beam at the level of the elbow would be expected to reduce beam-hardening and photon-starvation artifacts (8, 10, 11). Vali et al. (6) and Rohr et al. (7) similarly used elbow CT protocols in dogs with the head retracted to reduce superimposition and artifacts, in either lateral or sternal recumbency. To our knowledge, the specific variant used in the present study (a single thoracic limb extended cranially with the head and neck flexed caudally out of the scan plane) has not previously been the subject of a peer-reviewed methodology study. Our findings therefore provide the first systematic quantitative and qualitative evaluation of this variant against dorsal recumbency with the head in neutral position, and demonstrate that the artifact reduction is both objectively measurable and visually obvious to trained observers.

Contrary to our initial hypothesis, doubling the tube current from 150 mA to 300 mA did not produce a statistically significant change in SNR or CNR within either recumbency: the within-recumbency dose comparisons did not reach statistical significance for any outcome. This is consistent with the well-established relationship between tube current and image noise in CT, in which proportional increases in mAs yield only square-root improvements in SNR—doubling the dose increases SNR by approximately 40% rather than 100% (9). Observer preferences corroborated this pattern: for bone-window conspicuity, the higher tube current was preferred over the lower in approximately one-third of paired comparisons within each recumbency, but most paired comparisons (~63%) were rated as ties, indicating limited perceptible benefit of doubling the dose once a given recumbency was used. The clinical implication is that 150 mA at 100 kVp provides diagnostic image quality under the protocol used here. The modest additional benefit of 300 mA can be reserved for selected clinical contexts in which photon starvation is expected to be severe; for example, in very large or obese dogs where the tissue path length at the elbow is substantially increased, or in dogs with metallic orthopedic implants where photon-starvation and metal-related streak artifacts are anticipated (10, 11).

A key clinical question is whether suboptimal positioning can be compensated by increasing the radiation dose. Our pre-planned cross-comparison answered this directly: lateral recumbency at 150 mA produced significantly higher SNR and CNR than dorsal recumbency at 300 mA for all four outcomes. Doubling the dose in dorsal recumbency therefore did not recover the image quality achievable with appropriate positioning at half the dose. From a radiation-protection perspective, this argues strongly that positioning should be optimised before tube current is increased (9, 14). Qualitative findings reinforced the quantitative conclusion: observers strongly preferred lateral recumbency at 150 mA over dorsal recumbency at 300 mA for the artifact criterion and for soft-tissue conspicuity. For bone-window conspicuity, however, no observer preference was observed between the two conditions, indicating that observers could not distinguish bone conspicuity between optimal positioning at low dose and suboptimal positioning at high dose—a finding that fits the broader pattern of partial dissociation for this criterion.

Although objective measurements showed an approximately two-fold increase in bone-kernel SNR and CNR for lateral compared with dorsal recumbency, the corresponding observer preference was modest and accompanied by a high tie rate. Across 240 observer-comparisons at 150 mA in the full group, lateral recumbency was rated superior in 41.7% of comparisons, dorsal recumbency in 27.1, and 31.2% were rated as ties. The non-tie margin in favour of lateral (60.6%) was statistically significant (sign test *p* = 0.008) but small in absolute terms, and the high tie rate indicates that nearly one in three paired comparisons produced no detectable subjective difference despite the objective difference in the same elbows. By contrast, for the artifact criterion and soft-tissue conspicuity, observer preferences were near-unanimous in favour of lateral recumbency, matching the quantitative findings. This partial dissociation between SNR/CNR improvement and subjective bone-conspicuity preference is consistent with established CT physics (8). Edge sharpness for high-contrast structures, such as the cortical margin of the medial coronoid process or the trabecular pattern of cancellous bone against marrow, is governed primarily by the spatial-resolution chain (focal-spot size, slice thickness, reconstruction kernel and pixel size), not by signal- or contrast-to-noise ratio (8). SNR and CNR govern the detectability of low-contrast structures, not the perceived sharpness of structures whose attenuation difference from their surroundings already greatly exceeds the noise floor (8, 15). Because the spatial-resolution parameters were identical between dorsal and lateral acquisitions, the additional SNR available in lateral recumbency translated into a perceptible bone-conspicuity advantage only intermittently. We note that streak artifacts crossing the medial coronoid process would also be expected to degrade perceived sharpness, and their reduction under lateral recumbency should therefore have contributed to improved bone conspicuity. Interestingly, this did not consistently translate into observer preference and makes the subjective-objective dissociation even more noteworthy.

This phenomenon, in which objective image-quality metrics continue to scale beyond the level of perceptible benefit, has been highlighted in recent reviews of CT image quality assessment, which emphasise that ROI-based SNR and CNR do not fully capture the perception of radiologists (15). Inter-observer agreement for visual interpretation of subtle CT features of the medial coronoid process is also known to be modest, given the considerable normal anatomical variability at this site (4). The presence of pre-existing medial coronoid disease and other degenerative changes in many of the cadavers may be considered an advantage of this study, as it more closely reflects the clinical population in which elbow CT is routinely performed. The presence of pre-existing pathology had a more nuanced effect on bone-window conspicuity ratings than initially anticipated: inter-elbow variability in trabecular pattern and medial coronoid margin may have added noise to the conspicuity scoring in some elbows, while focal degenerative lesions (osteophytes, enthesophytes and medial coronoid fragmentation) in others were actually helpful, providing observers with well-defined lesion margins to compare across recumbencies that were absent when only smooth trabecular bone was available (2). The persistence of inconsistent observer preferences even in elbows where such focal lesions provided clear comparison anchors further supports a perceptual rather than an anatomic explanation for the dissociation. In any case, neither effect is likely to be the principal explanation, since the same elbows produced unanimous artifact-criterion scores.

The clinical implications of our findings depend on the diagnostic question being asked. For routine orthopedic CT of the canine elbow, where the primary aim is assessment of the medial coronoid process and adjacent cortical structures, dorsal recumbency remains a reasonable choice. The observer-perceived advantage of lateral recumbency for bone-window conspicuity was real but modest at the group level, with nearly a third of paired reads showing no perceptible difference. Therefore, dorsal recumbency does not entail a clear penalty for the observer-perceived sharpness of high-contrast bony structures. Dorsal recumbency also offers several practical advantages: both elbows are imaged in a single acquisition rather than the two separate acquisitions required for lateral recumbency, reducing total scan time, cumulative radiation dose and, in clinical practice, anaesthesia duration (9); the symmetrical positioning of both forelimbs allows direct side-by-side comparison of the left and right elbows at matched anatomical levels, particularly useful given the high prevalence of bilateral elbow dysplasia (1, 3); and the same acquisition can, when clinically indicated, be extended to include the shoulders, brachial plexus and cervical spine for cases of difficult-to-localise forelimb lameness. Lateral recumbency should be preferred when artifact reduction is clinically important (for example, in very large or obese dogs, in dogs with contralateral orthopedic implants—an extrapolation, as implants were an exclusion criterion in this study—or in second opinion cases where artifact reduction is critical to interpretation), or when detailed evaluation of periarticular soft-tissue forms part of the diagnostic question, where observer preferences were near-unanimous in its favour. The cross-comparison further argues that, when lateral recumbency is selected, 150 mA at 100 kVp is sufficient for diagnostic image quality, in keeping with the ALARA principle (14).

### 4.1 Limitations

This was a cadaver study. Patient motion, breath-hold artifacts, and physiological tissue conditions of live patients are not investigated and may impact the image quality in clinical practice. Findings are therefore preliminary and could be confirmed in a live-patient cohort.

The subgroup analyses (*n* = 8 cadavers, 16 elbows) had a small sample size (14, 16). The chosen subgroup size is nonetheless comparable to that used in previous veterinary CT image-quality studies [Lee et al. (14) *n* = 10 dogs; Davies et al. (16) *n* = 10 cadaver heads]. Wilcoxon signed-rank tests on paired observations within elbow were used, but possible non-independence of paired elbows within cadaver was not formally modelled. The small sample size also limits power to detect modest effects, particularly for the within-recumbency dose contrasts; the absence of a statistically significant dose effect should therefore be interpreted as a non-detection rather than proven equivalence.

Complete blinding to acquisition condition was not achievable for recumbency in either group, and this is a principal limitation of the qualitative analysis. Partial visibility of the CT table within the imaged field allowed recumbency to be inferred from the image itself in both groups, despite anonymisation and randomised reading order. No procedural cue to the acquisition condition was given to the observers, but the possibility that observer preferences for the recumbency factor were influenced by knowledge of position cannot be excluded, and the qualitative findings for the recumbency factor should be interpreted with this caveat in mind. By contrast, observer preferences for the tube-current factor in the subgroup are not subject to this caveat, because tube current produces no visible cue on the image analogous to the CT-table visibility.

Results may not generalise to other scanner manufacturers, detector geometries, reconstruction kernels, or tube voltages. The choice of 100 kVp was clinically motivated for our institutional protocol; lower voltages may behave differently with respect to beam-hardening artifacts (10, 11).

### 4.2 Future research

Confirmation of these positioning effects in a live-patient cohort could be a follow-up study. Other useful extensions include evaluation at lower tube voltages to investigate the dose-contrast trade-off, and assessment of whether the positioning effect is similar in brachycephalic or smaller breeds where anatomical proportions of the thorax and forelimbs differ from the medium- to large-breed dogs included here.

A key question this study does not directly address is whether the observed differences in image quality translate into differences in diagnostic accuracy for detection and characterisation of elbow pathology. Image-quality endpoints such as SNR, CNR and subjective image-quality preferences are surrogate measures whose relationship to clinical diagnostic performance is not straightforward—particularly for bone-window conspicuity, where the partial subjective-objective dissociation we observed could potentially correspond to differences in diagnostic outcomes. Dedicated diagnostic-accuracy studies in a clinical screening population, comparing sensitivity, specificity and inter-observer agreement for the detection of medial coronoid disease and other elbow pathologies across recumbencies and tube currents, are needed to determine whether the image-quality advantages demonstrated here translate into meaningfully better diagnostic outcomes.

Finally, additional positioning protocols are used in clinical practice—including sternal, dorsal or lateral recumbency with both thoracic limbs positioned next to each other and the head pulled caudally out of the gantry, permitting single-acquisition imaging of both elbows. Systematic comparison of these protocols with the two evaluated here could be a valuable extension of this work.

## 5 Conclusion

Lateral recumbency reduces beam-hardening and photon-starvation artifacts and improves objective SNR and CNR for CT of the canine elbow by approximately two-fold, a margin that cannot be matched by doubling the tube current in dorsal recumbency. Observer preferences confirmed the lateral advantage for the artifact criterion and for soft-tissue conspicuity but were modest and inconsistent for bone-window conspicuity. Lateral recumbency is therefore preferred when artifact reduction is clinically important or when detailed soft-tissue evaluation is required. For routine orthopedic elbow CT focused on the medial coronoid process and adjacent cortical structures, dorsal recumbency remains acceptable and offers practical advantages of single-acquisition imaging of both elbows, symmetrical anatomy for left–right comparison, reduced scan and anaesthesia time, and the option to include the shoulders, brachial plexus and cervical spine when clinically indicated. Doubling the tube current from 150 to 300 mA did not produce a statistically significant within-recumbency improvement and did not recover image quality lost to suboptimal positioning. Following the ALARA principle, 150 mA at 100 kVp provides diagnostic image quality under the protocol used here.

## Data Availability

The raw data supporting the conclusions of this article will be made available by the authors, without undue reservation.
